# Multiple metastases of soft tissue visualized by technetium-99m-methylene diphosphonate scintigraphy: a case report

**DOI:** 10.1186/1752-1947-8-459

**Published:** 2014-12-22

**Authors:** Mauro Liberatore, Valentina Megna, Gregorio Patrizi, Domenico Giannotti, Camilla Proietti Semproni, Stefania Rebonato, Flavio Barchetti, Pietro Gallo, Giandomenico Miscusi

**Affiliations:** Department of Surgical Sciences, “Sapienza” – University of Rome, Viale Regina Elena 324, 00161 Rome, Italy

**Keywords:** BMP, Colorectal cancer, Heterotopic bone formation, Metastatic disease, Ossification nidus, Scintigraphic bone scan, Soft tissue metastasis

## Abstract

**Introduction:**

Lungs and liver are the most common sites of colorectal cancer metastases after regional lymph nodes, but metastases to unusual sites are reported. Heterotopic bone formation in soft tissues from colorectal cancer is a rare metastatic occurrence.

**Case presentation:**

We present the case of a 29-year-old Caucasian man affected by colon adenocarcinoma with multiple soft tissue metastases visualized by magnetic resonance imaging, computed tomography scan and scintigraphic bone scan. We suppose that these findings can be due to the fact that the tracer is concentrated in the ossification nidus of soft metastases.

**Conclusions:**

Our experience suggests that, in the presence of colon adenocarcinoma, a bone scan could be a sensible tool to highlight bone lesions or heterotopic bone nidus in soft tissues and that any subcutaneous lesion should be resected to avoid underestimating a potential malignancy.

## Introduction

The clinical presentation of colorectal cancer is usually related to abdominal symptoms, even if peculiar presentations are reported [1, 2], and the metastatic process usually involves at first regional lymph nodes [3], then liver and lungs [4]. However, the literature presents some cases of colorectal cancer with unusual sites of metastases such as skeletal muscle, heart, thyroid, adrenals, spleen, ovary, brain and larynx [5, 6]. Heterotopic bone formation occurs occasionally in colorectal polyps, Peutz Jeghers syndrome, Barrett’s esophagus, colorectal carcinoma, mucocele of the appendix and in abdominal laparotomic scars [7–9] but rarely occurs within metastatic tumor deposits [10–12]. We report the case of a young man with a curious symptomatic onset and unusual metastatic patterns from colon carcinoma.

## Case presentation

A 29-year-old Caucasian man, in good health until February 2010, underwent several diagnostic clinical examinations for dizziness and headache. He was therefore visited by different specialists who, assuming an inner ear disorder, recommended vestibular tests with no pathological results.

While he was undergoing all the tests to investigate the origin of his headache, he noticed two oval hard subcutaneous masses in his left lower limb, one in his thigh and the other in the lower third of his leg. According to his doctor, he underwent a specific ultrasound examination which demonstrated the presence of nodular hyperechoic inhomogeneous lesions with posterior attenuation and the cleavage plane with the bone surface could not be evaluated.

For this reason a magnetic resonance imaging of his leg was performed and showed that the oval structure presented a diffusely hyperintense signal on T1-weighted (1.8×1cm) in his peroneal muscles, which was connected by a narrow bridge (4×2mm) to the extensor digitorum longus and the bone surface was preserved.

He then underwent surgical exeresis of the fusiform lesion in the peroneal muscle of his left leg.

Histology demonstrated macroscopically a grey-brown lesion (3×1.5×1cm); after it was cut a whitish mass was observed with well-defined margins and yellowish streaking. Microscopic examination showed the presence of large necrotic areas of infiltration of striated muscle and bone tissue which was marked by alcian blue on the margin of the resection. The pattern of the fusiform mass was that of an unspecified adenocarcinoma. There were no deposits of mucin; these elements could not suggest the origin so his lung was then suspected as the source.

Immunohistochemical examination was mandatory. The neoplastic cells were negative for thyroid transcription factor 1 (TTF-1) and cytokeratin (CK) 7, whereas they were positive for carcinoembryonic antigen (CEA) and CK20 and the protein encoded by the caudal type homeobox 2 (CDX2), therefore suggesting a colonic source contrary to the initial suspicion of lung adenocarcinoma.

He underwent blood tests which showed in particular neutrophilia, increased alpha amylase levels (162U/L), lipase 247UI/L, fibrinogen (589mg/dL), CEA 270 (<5ng/mL), tissue plasminogen activator>4000 (<85U/L), carbohydrate antigen (CA) 19–9 (gastrointestinal cancer antigen)>210 (<37IU/mL), beta-human chorionic gonadotropin 0.60 (<5mU/mL), CA72-4>100 (<37U/mL), neuron-specific enolase 29 (<10ng/mL) and CK fragment>62 (<3.3ng/mL).

**Figure 1:**
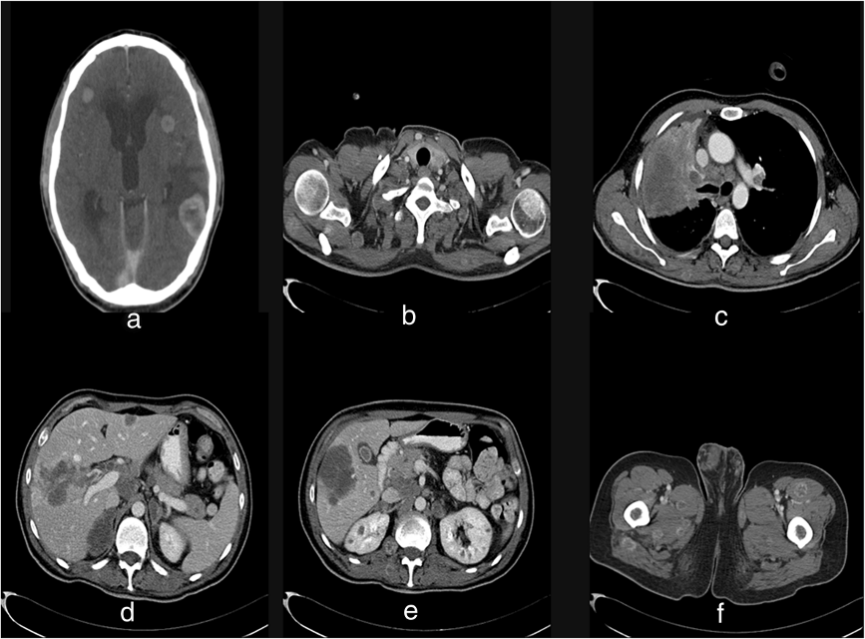
**Whole body computed tomography. a)** secondary lesions of the brain; **b)** multiple nodules in the muscles of shoulder joint; **c)** presence of a large mass (about 9×10×9cm), not homogeneous, partially necrotic, occupying the upper half of the right hemithorax, with atelectasis of upper and medium lobes. Multiple nodules with irregular margins and some groups of pathological lymph nodes were located in the mediastinum; **d)** 10 to 12 secondary lesions in the liver parenchyma; **e)** similar lesions in the pancreas, adrenal glands and kidneys; **f)** several hypervascularized lesions in the muscles of the upper third of the lower limbs.

He then underwent a whole body computed tomography (Figure 1) (CT) scan that showed at least 25 secondary lesions in his brain and cerebellum, a large mass (about 9×10×9cm), inhomogeneous, partially necrotic, occupying the upper half of his right hemithorax and determining complete atelectasis of upper and medium lobes of his right lung, and multiple thoracic nodules with irregular margins and ranging in size from 4 to 16mm. Multiple lymphadenopathies were evident at right and left hilum, in his mediastinum and in his neck. In the hepatic parenchyma there were 10 to 12 confluent metastatic nodules and other similar lesions in his pancreas, adrenal glands and kidneys. A neoplastic mass invading the full thickness of the wall of his sigmoid colon was demonstrated. Several hypervascularized secondary nodules were observed in most of the examined muscles, particularly in his pelvic and scapular joints. No evidence of pathological changes was found in his bones.

**Figure 2:**
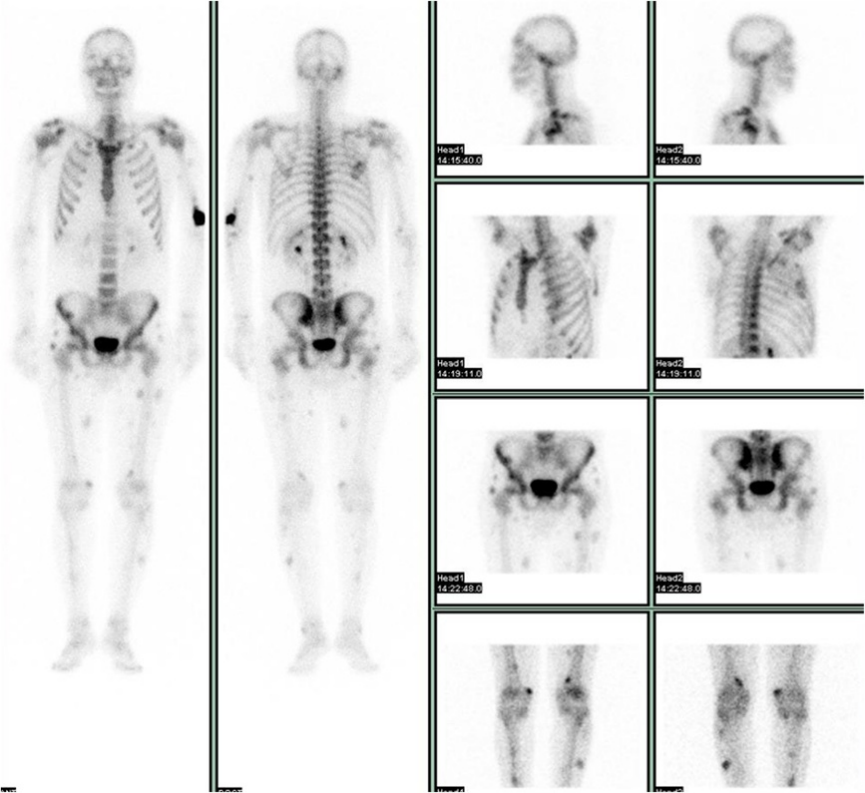
**Bone scan.** Different areas of concentration of the radiopharmaceutical are evident in the soft tissues of the upper and lower limbs and of the pelvis, while the skeletal tissue is undamaged.

Meanwhile, he underwent a whole body scintigraphic scan (Figure 2) that showed several areas of increased uptake of osteotropic tracer in soft tissues, especially in his lower and upper limbs and in his pelvis without evidence of increased uptake in his bones. These areas of hyperfixation of osteotropic tracer corresponded to the nodules observed in the CT scan.

Afterwards he started chemotherapy and whole brain irradiation. Despite the treatment, he worsened fast and became aphasic and his relatives decided to stop treatment. He died 15 days later after a cardiac arrest.

## Discussion

Heterotopic bone formation is more likely to occur with gastrointestinal cancers and their metastases, especially the mucin-secreting variety, rather than other epithelial tumors [7, 8, 10, 13] and the rectum is the most common site [14].

Recent studies suggest that the transformation of pluripotent mesenchymal cells into osteoblasts causes the formation of bone tissue in skeletal muscular tissue [10, 12]. It is still unclear which factors influence the transformation of mesenchymal cells. The ossification process is prevalently observed in tumors that secrete mucin [7, 13, 14]. In a case report [15] by Naik *et al*. in 2005, a metastatic intramuscular mass removed from the suprapubic area of a 56-year-old man with rectal adenocarcinoma turned out at histology to be a lesion without muscular tissue made of nidus of mature bone tissue with osteoblasts and osteocytes alternated with necrotic areas and deposits of mucin with no bone marrow formation or cartilage tissue. This suggests that malignant tumors secreting mucin can produce a substance which stimulates the differentiation of mesenchymal cells into osteoblasts and that neoplastic necrosis provides a nidus for metaplastic ossification [8, 15]. Randall *et al*. describe the case of a 69-year-old man with adenocarcinoma of the transverse colon that developed heterotopic ossification in a metastatic axillary lymph node. Immunostaining for alkaline phosphatase revealed a significant concentration of this enzyme in these cells and, to a lesser degree, on the apical membrane of the glandular cells of the adenocarcinoma adjacent to the ossification centers, concluding that alkaline phosphatase is intimately associated with bone formation under these pathologic conditions [11].

Hobdy *et al*. describe a case of two unusual metastatic lesions, one in the soft tissues of the chin and the other in the psoas muscle in a man with an adenocarcinoma of the rectum. Immunostaining of the first lesion was positive for CEA and CK20 but negative for CK7 [4].

In the present case, traditional histology was not conclusive and immunostaining was positive for CEA, CK20 and CDX2 and negative for TTF-1 and CK7. In a study on heterotopic ossification from colon adenocarcinoma, Imai *et al*. reported the presence of bone morphogenetic proteins (BMP), specifically BMP5 and BMP6, in the cytoplasm of tumor cells and weak staining in osteoblast-like cells adjacent to newly formed bone. BMP2 and BMP4 are less expressed in tumor cells, osteoblast-like cells and stromal cells [16]. These immunohistochemical data suggest that tumor cells expressing BMP5 and BMP6 induce the transformation of mesenchymal cells, which normally express BMP2 and BMP4, in preosteoblasts and osteoblasts [8].

A new BMP (BMP9) has been recently identified and shown to be the most potent BMP [17]. Leblanc *et al*. [18] first demonstrated that BMP9 could efficiently induce the osteogenic program of muscle-resident mesenchymal or stromal cells. Of interest, the injection of BMP9 straight into muscles does not induce ossification unless the muscle is damaged, indicating that cells in regenerating tissue are more permissive to environmental cues than cells in resting tissue.

The concomitant bone scintigraphic positivity of metastasis in the soft tissue is also worthy of note. Bone scintigraphy is performed by intravenous injection of technetium-99m-methylene diphosphonate (99^m^Tc-MDP) and subsequent acquisitions of total body images after about 2 hours. The principal bonding mechanism of phosphate and phosphonates on the bone is chemical absorption which occurs through chemical bonds in the bending and dislocation on the surface of the hydroxyapatite crystals, while tin and 99^m^Tc are hydrolyzed and bound to the bone either separately or together in the form of tin dioxide and Tc-dioxide. The large surfaces of hydrated hydroxyapatite, present in the growth centers or in the metabolically active bone lesions, facilitate a greater chemical absorption and therefore show a greater capacity to concentrate the radiopharmaceutical. Increased uptake of the radiopharmaceutical occurs in areas involved by pathological phenomenon: the most important factor is represented by the blood flow which carries the radiopharmaceutical (in the case of pathological increased blood flow the uptake increases also); a second factor is an increase of metabolic activity in correspondence with the bone lesions (the fixation is proportional to the degree of bone turnover).

The hypervascularization of multiple soft tissue metastases without macro- or microcalcification evidenced by CT scan, their intense uptake in bone scintigraphy and positivity for CK20 and CEA, are suggestive of the presence of heterotopic bone formation in soft tissue from colon adenocarcinoma.

Unfortunately, the reported case must be interpreted in the light of certain limitations. We do not have the histologic data to prove that the tissue is heterotopic bone rather than dystrophic bone formation. Scintigraphic bone uptake was not totally unambiguous as showed in other studies [19]. Nevertheless, in the general context of this case, we are fairly confident that those foci were heterotopic bone formation and not just soft tissue calcifications.

## Conclusions

Our experience suggests that, in the presence of colon adenocarcinoma, a bone scan could be a sensible tool to discover not only bone lesions but also possible heterotopic bone nidus in soft tissue. Furthermore, any subcutaneous lesion should be resected as soon as possible to avoid underestimating a potential malignancy [20].

## Consent

Written informed consent was obtained from the patient’s next of kin for publication of this case report and any accompanying images. A copy of the written consent is available for review by the Editor-in-Chief of this journal.

## Abbreviations

99^m^Tc-MDP: Technetium-99m-methylene diphosphonate
BMP: Bone morphogenetic proteins
CA: Carbohydrate antigen
CDX2: Protein encoded by the caudal type homeobox 2
CEA: Carcinoembryonic antigen
CK: Cytokeratin
CT: Computed tomography
TTF-1: Thyroid transcription factor 1.
